# Early and intensive motor training to enhance neurological recovery in people with spinal cord injury: trial protocol

**DOI:** 10.1038/s41393-023-00908-z

**Published:** 2023-07-06

**Authors:** Lisa A. Harvey, Joanne V. Glinsky, Jackie Chu, Robert D. Herbert, Hueiming Liu, Stephen Jan, Laurent Billot, Giorgio Scivoletto, Annemie I. Spooren, Henk A. Seelen, Marsha Ben, Keira Tranter, Lydia W. Chen, Donna Rainey, Christine Rimmer, Vivien Jorgensen, Fernanda Di Natal, Sophie Denis, Emilie J. Gollan, Federica Tamburella, Jacqui Agostinello, Charlotte M. van Laake-Geelen, Chris Bell, Claire Lincoln, Janneke M. Stolwijk, Jessica van der Lede, Sue Paddison, Kristine Oostra, Ian D. Cameron, Gerard Weber, Catherine Sherrington, Andrew K. Nunn, Emma-Leigh Synnott, Euan McCaughey, Jasbeer Kaur, Sachin Shetty

**Affiliations:** 1 John Walsh Centre for Rehabilitation Research, University of Sydney, Kolling Institute, Sydney, NSW Australia; 2 Neuroscience Research Australia (NeuRA), Sydney, NSW Australia; 3 The George Institute for Global Health, University of New South Wales, Sydney, NSW Australia; 4 I.R.C.C.S. Santa Lucia Foundation, Rome, Italy; 5 REVAL, Hasselt University, Hasselt, Belgium; 6 Adelante Centre of Expertise in Rehabilitation and Audiology, Hoensbroek, The Netherlands; 7 Royal North Shore Hospital, Sydney, NSW Australia; 8 Royal Rehab, Sydney, NSW Australia; 9 Fiona Stanley Hospital, Perth, WA Australia; 10 Sunnaas Rehabilitation Hospital, Nesodden, Norway; 11 Prince of Wales Hospital, Sydney, NSW Australia; 12 The Princess Alexandra Hospital, Brisbane, QLD Australia; 13 The Royal Talbot Rehabilitation Centre, Melbourne, VIC Australia; 14 Department of Rehabilitation Medicine, Research School CAPHRI, Maastricht University, Maastricht, The Netherlands; 15 Repatriation General Hospital, Adelaide, SA Australia; 16 Queen Elizabeth National Spinal Injures Unit, Glasgow, UK; 17 Center of Excellence for Rehabilitation Medicine, University Medical Center Utrecht Brain Center, University Medical Center Utrecht, Utrecht, The Netherlands; 18 De Hoogstraat Rehabilitation, Utrecht, The Netherlands; 19 London Spinal Cord Injury Centre, Royal National Orthopaedic Hospital Trust, Middlesex, UK; 20 Ghent University Hospital, Ghent, Belgium; 21 Sydney School of Public Health, Faculty of Medicine and Health, University of Sydney, Sydney, NSW Australia; 22 Victorian Spinal Cord Service, Austin Health, Melbourne, VIC Australia

**Keywords:** Health care, Neurology

## Abstract

**Study design:**

Protocol for a multi-centre randomised controlled trial (the SCI-MT trial).

**Objectives:**

To determine whether 10 weeks of intensive motor training enhances neurological recovery in people with recent spinal cord injury (SCI).

**Setting:**

Fifteen spinal injury units in Australia, Scotland, England, Italy, Netherlands, Norway, and Belgium.

**Methods:**

A pragmatic randomised controlled trial will be undertaken. Two hundred and twenty people with recent SCI (onset in the preceding 10 weeks, American Spinal Injuries Association Impairment Scale (AIS) A lesion with motor function more than three levels below the motor level on one or both sides, or an AIS C or D lesion) will be randomised to receive either usual care plus intensive motor training (12 h of motor training per week for 10 weeks) or usual care alone. The primary outcome is neurological recovery at 10 weeks, measured with the Total Motor Score from the International Standards for Neurological Classification of SCI. Secondary outcomes include global measures of motor function, ability to walk, quality of life, participants’ perceptions about ability to perform self-selected goals, length of hospital stay and participants’ impressions of therapeutic benefit at 10 weeks and 6 months. A cost-effectiveness study and process evaluation will be run alongside the trial. The first participant was randomised in June 2021 and the trial is due for completion in 2025.

**Conclusions:**

The findings of the SCI-MT Trial will guide recommendations about the type and dose of inpatient therapy that optimises neurological recovery in people with SCI.

**Trial registration:**

ACTRN12621000091808 (1.2.2021).

## Introduction

Any neurological recovery following spinal cord injury (SCI) can improve mobility, independence and quality of life for people with SCI. Recovery of motor function is particularly important. This is most commonly quantified by changes in the total motor score that forms part of the International Standards for Neurological Classification of SCI [1–4]. Many different physical rehabilitation interventions and approaches have been developed and advocated over the years in an effort to promote neurological recovery and improve total motor scores [5, 6]. However, to date, no clinical trial has examined the effect of an intensive individualised programme of task-specific training supplemented with strength training provided soon after injury. We refer to this intervention as—“motor training” directed at and below the level of injury.

Task-specific training was probably first advocated in a rehabilitation context by Carr and Shepherd in the 1980s [6]. They proposed the use of task-specific training for rehabilitation after stroke. However, it has also been used in the rehabilitation of people with SCI since this time [7, 8]. Task-specific training involves the training of any functional activity such as sitting, moving from sitting to standing, standing and walking, as well as grasping and manipulating objects with the hands [9]. It relies on therapists using clinical reasoning to identify deviations from normal movement patterns and then designing individualised training programmes that typically consists of part- and whole-practice. Key features of task-specific training include goal setting, feedback and progression with an emphasis on repetitious practice [10–13]. It can be augmented with the judicious use of biofeedback, electrical stimulation, robotics and computer game-based therapy but the use of technology alone does not define it. Importantly, task-specific training does not include passive therapies such as manual therapy, vibration, joint mobilisations, passive standing, massage or stretching.

Dosage is a key consideration for any therapy for all types of neurological conditions but particularly for task-specific training where repetitious practice is at its core [11, 12, 14–17], and particularly if trying to prompt an injured spinal cord to repair itself. The need to increase the intensity of therapy was initially highlighted in 1990s by the work of Kwakkel but in the area of stroke rehabilitation where the focus is on prompting recovery of the damaged brain [18]. However, the principles and key learnings are the same [19], and the need to increase the opportunity for practice has in part lead to the evolution of interventions such as treadmill training and robotics [20]. They all provide a way of increasing repetitious practice. Yet surprisingly, the clinical trials involving people with SCI that have included measures of neurological recovery (i.e., strength of neurologically-weak muscles) have not provided therapy in particularly high dosages. For example, our own systematic review that identified 26 clinical trials [21] found that treatments ranged from 0.5 to 3 h, 3 times per week (mostly for 4–6 weeks although for 24 weeks in one trial). Similarly, observational studies suggest that people undergoing rehabilitation following SCI have limited opportunity for repetitious practice. For example, an observational study from Canada in people with SCI undergoing rehabilitation found that people with tetraplegia only performed a median of 42 attempts at moving their upper limbs each day during both physiotherapy and occupational therapy at the time of admission to rehabilitation [22]. This dropped to 15 by the time of discharge. The situation was similar for the lower limbs in all patients. This same study indicated that people received a total of 1.5 h of physiotherapy and occupational therapy a day but less than 1 h of this time was devoted to moving the upper or lower limbs in any way. A similar comprehensive study involving SCI units from Norway, Australia and Netherlands painted a similar situation with patients receiving a median of 3.8 to 5 h of therapy per week at the SCI units in the three countries [23, 24]. It is not clear how much of this time was devoted to repetitious practice and repeated attempts at movement but it would have been considerably less than 3.8 to 5 h per week because the time captured included all aspects of therapy including education, assessment, and wheelchair skill training. These findings are not unique to SCI rehabilitation. Very similar observations have been made in stroke units around the world [25, 26]. Of course the situation is changing both with stroke and spinal cord injuries, and there are many initiatives to increase therapy time and the opportunities for task-specific practice [27, 28]. However, the effectiveness of these initiatives for people with SCI is yet to be proven in a high-quality clinical trial; the motivation for the SCI-MT Trial. The SCI-MT trial attempts to provide people with as much task-specific training on top of usual therapy that it is conceivably possible soon after injury, namely 12 h per week for 10 weeks commencing within 10 weeks of injury. The task-specific training will be supplemented with strength training because clinical trials involving people with SCI point to its effectiveness [21, 29–31], and because strength is core to any attempts at movement [32, 33]. The SCI-MT Trial includes people with some preserved motor function below the motor level (for those with an American Spinal Injuries Association Impairment Scale (AIS) A lesions) or with motor incomplete lesions (AIS C and AIS D). These individuals are being targeted because they are most likely to respond to the intervention [19].

In all, the aim of the Early and Intensive Motor Training for SCI trial (SCI-MT trial) is to determine the effect of early and intensive motor training on neurological recovery of people with SCI. Secondary aims are to determine the effects of this intervention on function, ability to walk, quality of life, participants’ perceptions about ability to perform self-selected goals, length of hospital stay and participants’ impressions of therapeutic benefit. A cost-effectiveness analysis and process evaluation will be run alongside the trial. This paper describes the protocol of the SCI-MT Trial.

## Methods

### Design

An international, multi-centre, investigator-initiated, pragmatic, randomised controlled trial will be undertaken. The first participant was randomised on 7th June 2021 and the trial is due for completion in 2025. The protocol was prospectively registered with the Australian New Zealand Clinical Trials Registry (ACTRN12621000091808).

### Participants

Two hundred and twenty participants will be recruited from 15 hospitals across Australia (7 sites), Scotland (1 site), Italy (1 site), England (1 site), Norway (1 site), Netherlands (2 sites) and Belgium (2 sites). The primary eligibility criterion is an American Spinal Injuries Association Impairment Scale (AIS) A lesion with motor function more than three levels below the motor level on one or both sides, or an AIS C or D lesion, sustained in the preceding 10 weeks. Participants will only be eligible if they are likely to remain an inpatient at the hospital for 10 weeks. The full inclusion and exclusion criteria are provided in Table 1.

**Table 1:** Inclusion and exclusion criteria for the SCI-MT trial.

Inclusion criteria	Exclusion criteria
1. Have sustained a traumatic or non-traumatic SCI of any neurological level in the preceding 10 weeks. 2. Have an AIS A lesion with motor function more than three levels below the motor level (on one or both sides), or an AIS C or AIS D lesion (as defined by the International Standards for the Neurological Classification of SCI). 3. Are male or female, over the age of 16 years at the time of signing informed consent (additional consent from a parent or guardian will be attained for those aged under 18 years).^a^ 4. Have been cleared by the medical team to commence rehabilitation (as documented in the participant’s medical files). 5. Are likely to remain an inpatient for the next 10 weeks. 6. Understand and voluntarily sign an informed consent form prior to any study related assessments/procedures being conducted. 7. Are willing and able to adhere to the study visit schedule and other protocol requirements.	1. Have any significant medical or physical condition (including pregnancy) or psychiatric illness that could prevent the person from participating in the trial, or would place the person at an unacceptable risk if he/she were to participate. 2. Are unable to provide informed consent. 3. Are family members of the research staff. 4. Are participating in another clinical trial that involves motor training of the key muscles (as defined by the International Standards for Neurological Classification of SCI). 5. Do not speak the national language.

^a^Two sites in Europe will not include 16 or 17 years olds because of regulations around the inclusion of people this age.

### Assignment of intervention

A blocked randomisation schedule stratified by site and severity of injury (paraplegia or tetraplegia) that incorporates random permuted blocks has been computer-generated by an investigator who is not involved in the day-to-day running of the trial. The schedule was uploaded onto REDCap with strict control of user rights to ensure concealment from potential participants, trial staff and investigators.

Randomisation will occur after consent has been given and the baseline assessment has been completed. The assessor will send an email request for allocation, whereupon a researcher who is otherwise not involved in the trial will log into the REDCap database, triggering the randomisation and sending of an automated email notification to the participant’s site. Allocation will be to one of two groups: a Motor Training Group or a Usual Care Group. Both groups will continue to receive usual physiotherapy and rehabilitation.

### Interventions

#### Usual care

In keeping with the pragmatic orientation of the trial, both groups will receive the physiotherapy and occupational therapy typically provided to inpatients at the site [24]. It is anticipated that for most sites this will be 3 to 6 sessions per week of both occupational therapy and physiotherapy. Some sites may also provide group classes, hydrotherapy and sessions with exercise physiologists. At some sites, usual care could include some components of the motor training intervention.

The amount and type of usual care at each site will be recorded. Specifically, the following variables will be collected: the date, time and length of both scheduled and delivered physiotherapy and occupational therapy sessions. We will differentiate scheduled and delivered sessions because scheduled sessions are often missed due to illness, medical appointments and other reasons. In addition, the time spent on seven broad types of commonly administered therapies [34] will be collected using the International SCI Physical Therapy-Occupational Therapy Basic Data Set [4].

#### Motor training

Participants allocated to the Motor Training Group will receive, in addition to usual care, an extra 12 h of motor training each week for 10 weeks. This will commence within two business days of randomisation. The 12 h per week of motor training can be provided at any time although it is anticipated that most sites will administer the additional motor training over five 2-h sessions on each weekday of the week. Some sites may provide the training in longer sessions on Saturdays and Sundays, or both. The therapy involves active, targeted motor training of neurologically-weak muscles below the level of the injury, and may consist of practice of functional activities using the principles of task-specific training, or isolated muscle contractions directed at improving strength or motor control. For example, a participant with some motor activity in the lower limbs will receive intensive task-specific training focusing on regaining the ability to sit, stand and walk. This could involve task-specific training of sitting, standing, gait training on a treadmill with bodyweight support or the use of robotic gait devices. A participant with some motor activity in the upper limbs and hands will receive intensive training of a range of reaching and grasping tasks. Training will be supplemented, where available and at the therapist’s discretion, with biofeedback, electrical stimulation, mental imagery and somatosensory stimulation. Robotics and computer game-based therapy will be used where available and appropriate provided they encourage active contraction of neurologically-weak muscles. Importantly, treatments will be individualised to the needs of each participant and follow key principles of task-specific training and strength training as outlined in a detailed intervention manual compiled for the trial. All training will address a participant’s specific motor problems. Details of the motor training provided to participants will be recorded with the same forms used to document usual care.

### Outcome measures

Outcome data will be collected at baseline (prior to randomisation), and then 10 weeks (±2 weeks) and 6 months (±4 weeks) after randomisation by independent blinded assessors. The baseline and 10-week assessments will be conducted face-to-face and the 6-month assessments will be conducted by telephone. For this reason, motor and sensory scores and AIS grades will not be collected at 6 months. The success of assessor blinding will be monitored and reported.

#### Primary outcome measure

The primary outcome is Total Motor Score (/100 points) 10 weeks after randomisation according to the International Standards for the Neurological Classification of SCI [2, 35].

#### Secondary outcome measures

The following 11 secondary outcomes will be collected (see Table 2 for the timing of each):

- American Spinal Injuries Association Impairment Scale [2, 35].
- Upper Extremity Motor Score [2, 35].
- Lower Extremity Motor Score [2, 35].
- Total Sensory Score [2, 35].
- Spinal Cord Independence Measure Version III—Self Report [36].
- Walking Index for Spinal Cord Injuries Version II [37].
- The World Health Organization Quality of Life—BREF [38] (the 4 domains will be analysed separately).
- EUROQOL—5D Health Questionnaire [39].
- Participants’ perceptions about ability to perform self-selected goals (based on the Goal Attainment Scale [40, 41] and the Canadian Occupational Performance Measure [42]).
- Participants’ impressions of therapeutic benefit [43].
- Time to discharge.

**Table 2:** The assessment schedule.

	Enrolment	Baseline	0–10 weeks	10 weeks	6 months
Preliminary data/processes					
Inclusion/exclusion criteria	✓		Intervention period		
Demographic details		✓			
Date of injury		✓			
Outcomes					
Total motor score		✓		✓	
AIS grade		✓		✓	
The Upper Extremity Motor Score		✓		✓	
The Lower Extremity Motor Score		✓		✓	
Total sensory scores		✓		✓	
SCIM-III		✓		✓	✓
WISCI-II		✓		✓	✓
WHOQOL-BREF (4 domains)		✓		✓	✓
The EQ-5D-5L		✓		✓	✓
Participants’ perceptions about ability to perform self-selected goals		✓		✓	✓
Participants’ impressions of therapeutic benefit				✓	✓
Time to discharge					✓

In addition, participants in the Motor Training Group will be asked to rate their perceptions of the benefits and harm from the intensive motor training. A second question will be asked about the burden of the intensive motor training using a similar format. Participants will be invited to provide any comments to help better understand their perceptions.

### Sample size

A sample size of 220 is needed to provide a 90% probability of detecting a significant between-group difference of 6/100 points on the Total Motor Score (/100 points) 10 weeks after randomisation. The decision to use 6/100 points is based on the recommendations of the Committee representing The International Campaign for Cures of Spinal Cord Injury Paralysis (ICCP) [44]. The committee states that 5 to 10 points reflects a “relatively modest treatment effect”. These calculations assume a SD of the change in Total Motor Scores of 13.3 points, a worst-case loss to follow-up in both groups of 10%, and an alpha value of 0.05. The estimated SD is based on comprehensive data from a large trial provided by the ICCP, and takes into account the exclusion of people with complete injuries and no zones of partial preservation, and the delayed recruitment for up to 10 weeks after injury (see Supplementary Fig. 2, pg 2, 2nd column, 3rd row in reference [44]). Allowance has been made for one interim analysis using the O'Brien-Fleming stopping rule.

### Data analysis

#### Primary outcome

The mean between-group difference in Total Motor Scores at 10 weeks will be estimated with a mixed linear regression model in which Total Motor Score at 10 weeks will be regressed on randomised group, baseline severity of injury (a stratification variable), and baseline motor score. Site (a second stratification variable) will be included as a random effect. Baseline scores will be included in the model to increase precision [45]. Severity of injury and study site will be included in the model to prevent the anti-conservative bias in standard errors that would otherwise be produced by stratification on these variables [46]. The effect of the intervention will be estimated as the adjusted mean between-group difference and confidence interval.

The primary analysis will be performed without replacing missing data. It will be expected to provide unbiased estimates of the intervention effect if data are missing at random conditional on non-missing data [47]. In case of non-negligible amounts of missing primary outcome data at 10 weeks (>5%), we will use controlled multiple imputations to assess under what conditions the results change, and how plausible these conditions are, using the approach described by Cro et al. [48].

A subgroup analysis will examine whether the effect of the intervention is moderated by the type of SCI at baseline (AIS A or AIS C/D), and severity of injury (paraplegia or tetraplegia). The subgroup analysis will be conducted on the primary outcome using a model that is the same as the linear regression model used in the primary analysis except that it contains, in addition, a term for the interaction between group and type of SCI.

#### Secondary outcomes

The effect of the intervention on upper and lower limb strength, sensation, independence, ability to walk, quality of life, perceptions of the ability to perform self-selected goals, and impressions of therapeutic benefit will be estimated using the same statistical model used in the primary analysis. For outcomes measured at both 10 weeks and 6 months, both visits will be included in the model and correlations between repeated measures will be modelled by including a random patient intercept and the effect of the intervention will be estimated at each visit by a fixed term for visit together with its interaction with the intervention.

Similar models will be used to estimate the effect of the intervention on participants’ impressions of therapeutic benefit. However, as baseline values are not collected for this outcome variable, baseline Total Motor Score will be used in the model as a pre-randomisation covariate.

The effect of the intervention on time to discharge will be estimated from a Cox model that includes baseline Total Motor Score and the stratification variables (severity as a fixed effect and site as a random effect). Patients will be censored at 6 months or when they were last known to be in hospital, whichever occurs earlier.

The effect of the intervention on type of SCI (AIS grade—5 levels) will be estimated using an ordinal logistic regression model that includes baseline AIS grade and the stratification variables. The effect of the the intervention will be estimated as the odds ratio corresponding to the odds of shifting to a worse grade category.

No subgroup, adjusted or imputed analyses are planned for any of the secondary outcomes.

If there is more than 20% non-adherence (defined as less than 80 h of additional motor training), methods of causal inference will be used to estimate the effects of intervention in the principal stratum of people who would comply with both assigned and (counterfactual) unassigned interventions [49]. Modern methods of causal mediation analysis will also be used to determine the extent to which the effects of the intervention are mediated by time spent in training [50, 51].

### Economic evaluation

Economic evaluations will be carried out from a health sector perspective. The components of resource use for the intervention that will be measured will include staff costs and consumables required to deliver the motor training. One-off costs such as equipment and the training materials will be amortised over an assumed life using standard discount rates. Separate evaluations will be conducted for each country using costs in local currencies obtained from study financial statements. As this intervention is being delivered on top of usual care, incremental costs can be estimated without costing usual care provided to all participants.

For those patients discharged during the study, additional health care use in terms of re-hospitalisations will be ascertained from study participants at follow-up in each of the study arms. Costs assigned to such hospitalisations recorded during follow-up will be based on published cost weights (for example, in Australia these will be sourced from the Independent Hospital Pricing Authority [52]).

At 6 months, health utilities derived from EQ-5D-5L scores (based on a relevant value set) will be multiplied by survival up to 6 months (although we expect deaths before 6 months to be very low). This will enable an estimate of QALY gain over 6 months for each participant, and in turn an estimate of the difference in average quality-adjusted life years between Motor Training and Usual Care Groups. The overall effectiveness of the intervention across the study will be used in each of the cost-effectiveness estimates. Set alongside country-specific incremental costs, an estimate of the incremental cost-effectiveness ratio will be derived for each country. Usual one way and probabilistic sensitivity analyses will be conducted to take into account uncertainty in estimates. The incremental cost-effectiveness ratio will be benchmarked against prevailing cost-effectiveness thresholds.

### Data integrity

Baseline and 10-week assessment data will be collected in paper format and transferred to George Clinical for double data entry into a REDCap database. There will be automatic checks for errors and data queries will be emailed to the sites and stored on the database. Electronically transcribed data will be managed by the Biostatistics and Data Management Division of George Clinical.

### Trial oversight and management

The trial will be overseen by a Steering Committee and a Management Committee. It will be managed by George Clinical: a Contract Research Organisation that has been commissioned on behalf of the sponsor to oversee the management of the trial.

### Site monitoring

Trial monitoring of the Australian sites will be performed by staff from George Clinical and by local monitors for sites outside Australia as specified in a detailed clinical trial monitoring plan.

### Data monitoring

An independent Data Monitoring Committee (DMC) will meet at least once per year to monitor the safety of trial participants and the quality of trial data. The DMC will be responsible for safeguarding interests of trial participants and protecting the integrity of the trial. The responsibilities and procedures of the DMC are detailed in a DMC Charter [53]. The DMC will conduct an unblinded interim analysis of efficacy and safety endpoints once 110 participants have completed the trial. The investigators and trial staff will remain blinded to the results of the interim analysis. The DMC may recommend continuing the trial, stopping the trial early, or modifying the trial. A recommendation to stop the trial early will be made only if there is clear evidence of a clinically important beneficial or harmful effect. The trial will not be stopped early on the grounds of futility.

### Adverse and serious adverse events

All participants will be screened weekly during the 10-week intervention period for adverse and serious adverse events. These will be reported to the lead Human Research Ethics Committee, Governance Office and sponsor as per local requirements. A serious adverse event is defined as any event that results in death, is life-threatening, requires inpatient hospitalisation or prolongation of existing hospitalisation, or results in a persistent or significant disability or incapacity. An adverse event is any untoward or unfavourable medical occurrence regardless of whether it is considered to be related to the participant’s involvement in the trial. However, adverse events that are highly prevalent in people with recent SCI and are unlikely to be related to the intervention (i.e., expected occurrences) will not be reported. These include urinary incontinence, urinary tract infection, respiratory infection and bowel incontinence (a full list of expected occurrences that will not be reported and as approved by the lead Human Research Ethics Committee is provided in the full protocol).

## Discussion

The SCI-MT Trial when completed will be one of the largest non-pharmaceutical investigator driven trials of a rehabilitation intervention in people with recent SCI. If the intervention is found to be effective, the results will be of immediate and direct relevance for inpatient care. However, the future potential rollout of the intervention will rely on the cost as well as overcoming any real or perceived barriers. For this reason, a detailed economic analysis and process evaluation will run alongside the trial.

The SCI-MT Trial is pragmatic. Consequently, it aims to mimic how intensive motor training would be administered in the real world. Some participants may not receive the full additional 12 h per week of motor training for 10 weeks for reasons outside their and our control. The reasons for departures from the scheduled intervention will be carefully recorded and accepted as part of the real-world situation and treatment paradigm for our primary intention-to-treat analysis. This is in keeping with the standard approach for pragmatic trials [54–58]. While the trial is pragmatic, we will nonetheless use contemporary causal modelling approaches to estimate the Complier Average Causal Effect of the intervention (as described in the “Methods” section).

We anticipate some variability in usual care. This will not confound estimates of the effect of the intervention. However, time spent in training may mediate the treatment effect [51]. For this reason we will determine the extent to which the effects of the intervention are mediated by time spent in training.

As of June 2023, 128 participants have been randomised (with 7 participants randomised per month). If the current rate of recruitment continues, the trial will be fully recruited by the end of 2024, as planned. Nonetheless, one of the biggest barriers to the success of any trial is recruitment [59]. In anticipation of this problem, the trial involves many sites around the world. Each site is required to recruit between 5 and 11 participants per year. This is considered achievable. If the rate of recruitment per month drops for any reason, we will consider opening more sites.

The Early & Intensive SCI-MT Trial will provide the first, rigorous evidence of the impact of a treatment approach designed to promote neural recovery and improve motor function following SCI. Improved neurological recovery and motor function could have tangible, meaningful and potentially life-altering implications for people with SCI. The results of this trial could also direct future guidelines for the inpatient rehabilitation care of these people.

## Supplementary information

AJ Checklist

## Data Availability

This is a trial protocol so there is currently no data to share.
